# SCORPION is a stacking-based ensemble learning framework for accurate prediction of phage virion proteins

**DOI:** 10.1038/s41598-022-08173-5

**Published:** 2022-03-08

**Authors:** Saeed Ahmad, Phasit Charoenkwan, Julian M. W. Quinn, Mohammad Ali Moni, Md Mehedi Hasan, Pietro Lio’, Watshara Shoombuatong

**Affiliations:** 1 Center of Data Mining and Biomedical Informatics, Faculty of Medical Technology, Mahidol University, Bangkok, 10700 Thailand; 2 Modern Management and Information Technology, College of Arts, Media and Technology, Chiang Mai University, Chiang Mai, 50200 Thailand; 3 Bone Biology Division, Garvan Institute of Medical Research, 384 Victoria Street, Darlinghurst, NSW 2010 Australia; 4 Faculty of Health and Behavioural Sciences, School of Health and Rehabilitation Sciences, The University of Queensland, St Lucia, QLD 4072 Australia; 5 Division of Biomedical Informatics and Genomics, John W. Deming Department of Medicine, School of Medicine, Tulane Center for Biomedical Informatics and Genomics, Tulane University, New Orleans, LA 70112 USA; 6 Department of Computer Science and Technology, University of Cambridge, Cambridge, CB3 0FD UK

**Keywords:** Computational biology and bioinformatics, Computational models, Machine learning

## Abstract

Fast and accurate identification of phage virion proteins (PVPs) would greatly aid facilitation of antibacterial drug discovery and development. Although, several research efforts based on machine learning (ML) methods have been made for in silico identification of PVPs, these methods have certain limitations. Therefore, in this study, we propose a new computational approach, termed SCORPION, (StaCking-based Predictior fOR Phage VIrion PrOteiNs), to accurately identify PVPs using only protein primary sequences. Specifically, we explored comprehensive 13 different feature descriptors from different aspects (i.e., compositional information, composition-transition-distribution information, position-specific information and physicochemical properties) with 10 popular ML algorithms to construct a pool of optimal baseline models. These optimal baseline models were then used to generate probabilistic features (PFs) and considered as a new feature vector. Finally, we utilized a two-step feature selection strategy to determine the optimal PF feature vector and used this feature vector to develop a stacked model (SCORPION). Both tenfold cross-validation and independent test results indicate that SCORPION achieves superior predictive performance than its constitute baseline models and existing methods. We anticipate SCORPION will serve as a useful tool for the cost-effective and large-scale screening of new PVPs. The source codes and datasets for this work are available for downloading in the GitHub repository (https://github.com/saeed344/SCORPION).

## Introduction

Bacteriophages are viruses that can infect and thrive in bacteria. It can be found in several environments including soil, freshwater and marine. The infectious phage particle is essentially comprised of a nucleic acid component (i.e. either DNA or RNA) in which they are encapsulated in a coat of protein known as capsids^1^. Individual types of bacteriophage can display an extremely high specificity towards a particular susceptible bacterial host species. The surface of which they will typically attach themselves irreversibly to and inject their genetic materials to the cellular interior. They are able to persist in the host by using one of two major strategies that are termed lytic and lysogenic life cycle^2^. Bacteriophages may represent a promising alternative to antiobiotics owing to the following properties: a lack of toxicity toward human cells, lack of harm caused to normal flora and ability to target antibiotic-resistant bacteria^3^. Phage structural proteins (PVPs) consists of capsid proteins, tail proteins and phage particle enzymes. PVPs are mainly responsible for orchestrating bacteriophage interaction with their specific bacterial hosts so their manipulation may represent an avenue to generate novel classes of antimicrobial agents^4^. Current experimental approaches for the identification of PVPs from non-PVPs include many scientific instruments and methodologies such as mass spectrometry, sodium dodecyl sulfate polyacrylamide gel electrophoresis (SDS-GE) based proteomic methods and protein analysis arrays^5–7^. While these methods represent gold standard approaches for PVP identification, they are difficult to employ for the analysis of PVPs at large scale as they are laborious and costly methods. Thus, researchers have invested much in efforts to develop computational models for predicting PVPs directly from their sequence information as a useful alternative.

To date, a variety of machine learning (ML)-based methods, including iVIREONS^8^, Feng et al.’s method^9^, PVPred^10^, PVP-SVM^11^, PhagePred^12^, Tan et al.’s method^13^, Ru et al.’s method^14^, Pred-BVP-Unb^15^ and PVPred-SCM^16^, Zhang et al.’s method^17^, Meta-iPVP^18^, iPVP-MCV^19^ and VirionFinder^20^ have all been developed and proposed for PVP identification. Table 1 provides a summary of these machine learning-based methods along with their employed ML algorithms, feature descriptors and evaluation strategies. In 2013, Seguritan et al. developed the first PVP predictor called iVIREONS^8^ based on ANN algorithm trained with AAC and PIP to predict viral structural proteins. Shortly afterward, Feng et al. created a high-quality dataset consisting of 99 PVPs and 208 non-PVPs, and also developed a NB-based predictor^9^ cooperating with AAC and DPC. Most recently, Han et al. developed an ensemble-based model named iPVP-MCV^19^ by combing three types of PSSM descriptors (i.e. PSSM-AAC, PSSM-composition and DP-PSSM). Until now, iPVP-MCV have represented a state-of-the-art predictor for PVP identification. More detail information for all of the existing methods is summarized in an article by Kabir et al.^21^. Although above mentioned methods do efficiently facilitate the prediction of PVPs, there are some issues that still need to be addressed. First, the training dataset used by several existing methods in PVP identification was relatively small. This is an important consideration, as several previous studies have demonstrated that training with a large number of datasets is crucial for building a comprehensive predictive model^18,22–24^. Second, almost all of the existing methods were developed by employing single ML methods to train the model. Therefore, their performance might not be optimal in some cases. However, ensemble models are capable to provide a greatly improved performance compared to baseline models^22,24–27^. Finally, the prediction performance for these existing methods is still not satisfactory for many real therapeutic applications.

**Table 1:** Characteristics of the existing methods for PVP prediction.

Predictors/tools	Year	Algorithm	Feature descriptors	Type	Evaluation strategy
iVIREONS^8^	2012	ANN	AAC, PIP	Single	10CV
Feng et al.’s method^9^	2013	NB	AAC, DPC	Single	10CV
PVPred^10^	2014	SVM	GGAP	Single	LOOCV, IND
Zhang et al.’s method^17^	2015	SVM	CTD, bi-profile Bayes, PAAC, PSSM	Ensemble	10CV, IND
PVP-SVM^11^	2018	SVM	AAC, ATC, CTD, DPC, PCP	Single	10CV, IND
PhagePred^12^	2018	NB	GGAP	Single	10CV, LOOCV
Tan et al.’s method^13^	2018	SVM	GGAP	Single	10CV, IND
Ru et al.’s method^14^	2019	RF	CCPA, AKSNG, Seq-Str	Single	10CV
Pred-BVP-Unb^15^	2019	SVM	CT, Bi-PSSM, SAAC	Single	LOOCV, IND
PVPred-SCM^16^	2020	SCM	DPC	Single	10CV, IND
Meta-iPVP^18^	2020	SVM	AAC, APAAC, DPC, CTDC, CTDD, CTDT and PAAC	Ensemble	10CV, IND
iPVP-MCV^19^	2021	SVM	PSSM-AAC, PSSM-composition and DP-PSSM	Ensemble	LOOCV, 10CV, IND
VirionFinder^20^	2021	CNN	AAI	Deep learning	10CV, IND
SCORPION	This study	RF	AAC, AAI, APAAC, CTDC, CTDD, CTDT, DDE, DPC, EAAC, PAAC, PSSM_AAC, PSSM_Com and PSSM_DP	Ensemble	10CV, IND

*ANN* artificial neural network; *CNN* convolutional neural network, *LR* logistic regression, *NB* naive bayes, *RF* random forest, *SCM* scoring card matrix, *SVM* support vector machine, *AAC* amino acid composition, *AACPCP* amino acid composition and physicochemical properties, *AKSNG* adaptive k-skip-n-Gram Algorithm, *APAAC* pseudo amino acid composition, *ATC* atomic composition, *Bi-PSSM* bigram position-specific scoring matrix, *CTD* composition translation and distribution, *DPC* dipeptide composition, *PSSM_DP* position-specific scoring matric based on dipeptides, *GGAP* g-gap dipeptide composition, *GGAPTree* g-gap feature tree, *PAAC* pseudo amino acid composition, *PCP* physicochemical properties, *PF* probabilistic features, *PIP* protein isoelectric points, *PSSM* position-specific scoring matrix, *PSSM_AAC* position-specific scoring matrix based on amino acid composition, *PSSM_COM* position-specific scoring matrix based on composition, *PSSM Profiles* position-specific scoring matrix based on profiles, *SAAC* split amino acid composition, *Seq-Str* sequence-structure, *10CV* tenfold cross-validation, *IND* independent test, *LOOCV* leave-one-out cross-validation.

**Figure 1:**
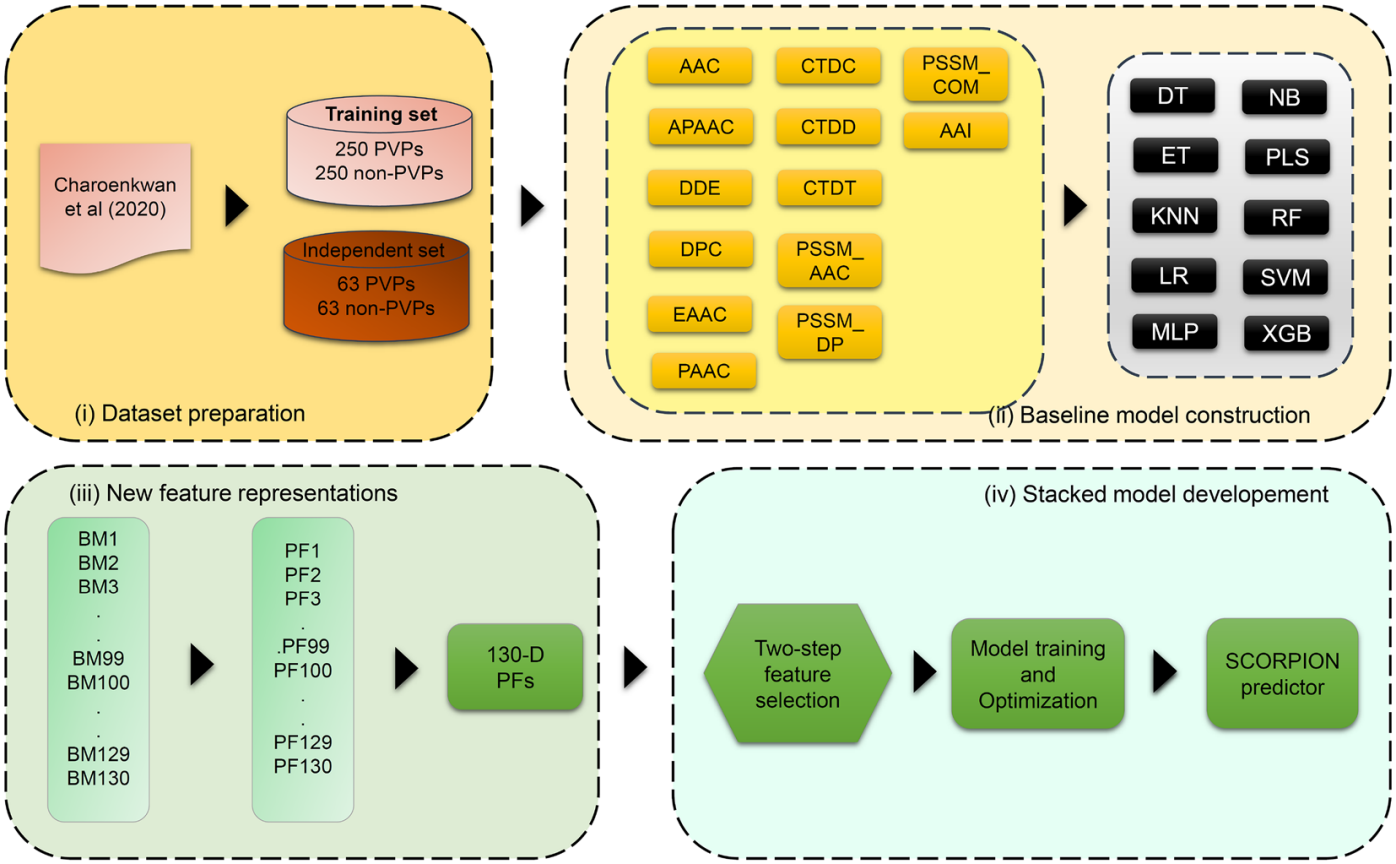
Schematic flowchart of the development of the SCORPION. It consists of dataset construction, baseline models construction, new feature representations and the stacked model development.

To address these limitations, we present a novel approach, termed SCORPION (StaCking-based Predictior fOR Phage VIrion PrOteiNs) to improve the accurate prediction of PVPs. The overall procedure for the development of SCORPION is illustrated in Fig. 1. Notably, SCORPION employs 13 different sequence-based feature descriptors from multiple perspectives (i.e., compositional information, composition–transition–distribution information, position-specific information and physicochemical properties) to extract the key pattern of PVPs. These feature descriptors were used to train a total of 130 baseline models by using 10 popular ML algorithms. Probabilistic features (PFs) were then generated by using these 130 baseline models, and considered as a new feature vector. To improve the predictive performance, a two-step feature selection strategy was applied to identify *m* out of 130 PFs. Finally, the optimal PF feature vector were used to develop an effective stacked model (SCORPION) by using the stacked ensemble learning strategy. Our comparative results base on cross-validation and independent tests indicate that SCORPION outperformed its baseline models. Moreover, SCORPION achieved a better performance than several existing methods for PVP prediction in terms of in terms of ACC (0.873), Sp (0.905), MCC (0.748) and AUC (0.891) on the independent dataset. These comparative results highlight the effectiveness and generalizability of SCORPION.

## Materials and methods

### Overall framework of SCORPION

As can be seen in Fig. 1, there exist four major steps, including dataset construction, baseline models construction, new feature representations and the stacked model development. First, The same benchmark dataset derived from Charoenkwan et al.^18^ were used to train and optimized baseline models and SCORPION. Second, 13 different feature descriptors were individually fed to 10 different ML algorithms to build the 130 baseline models using tenfold cross-validation. In addition, we comprehensively compared 13 different feature descriptors to determine the feature descriptors that are beneficial to PVP identification. Third, we constructed variant stacked models by using different sets of feature vectors. Forth, the optimal PF vector was determined and fed to RF algorithm in order to construct the final stacked model (SCORPION) by using the stacked ensemble learning strategy. Finally, we compared the predictive performance of SCORPION against its constitute baseline models and existing methods.

### Dataset collection

As described in an article by Kabir et al.^21^, there are three well-known benchmark datasets (i.e. Feng2013^9^, Manavalan2018^11^ and Charoenkwan2020_2.0^18^) that have been established for developing existing PVP predictors. In this study, we utilized the Charoenkwan2020_2.0 dataset established by Charoenkwan et al.^18^ as the benchmark dataset to assess the performance of SCORPION. Below, we provided two main reasons why we used the Charoenkwan2020_2.0 dataset. First, the Charoenkwan2020_2.0 dataset contained a larger number of PVPs and non-PVPs than other datasets. Specifically, the Charoenkwan2020_2.0 dataset combined Feng2013^9^ and Manavalan2018^11^ datasets along with novel PVPs and non-PVPs obtained from the UniProt database (release 2019_11)^28^. Second, a lower CD-HIT threshold of 0.4 was used to exclude more redundant sequences in the Charoenkwan2020_2.0 dataset. As a result, the Charoenkwan2020_2.0 dataset contained of 313 PVPs and 313 non-PVPs. In the Charoenkwan2020_2.0 dataset, the training and independent datasets (PVPs, non-PVPs) consisted of (250, 250) and (63, 63), respectively. All datasets used in this study are available on https://github.com/saeed344/SCORPION.

### Feature encodings

In this study, we used 13 different sequence-based feature descriptors containing amino acid composition (AAC), amino acid index (AAI), amphiphilic pseudo-amino acid composition (APAAC), composition in CTD (CTDC), distribution part of CTD (CTDD), transition in CTD (CTDT), dipeptide deviation from expected mean (DDE), dipeptide composition (DPC), enhance Amino Acid composition (EAAC), pseudo amino acid composition (PAAC), PSSM_AAC, PSSM_DP and PSSM_COM to extract the key information of PVPs and non-PVPs. These sequence-based feature descriptors provides us four different aspects consisting of compositional information, composition-transition-distribution information, position-specific information and physicochemical properties having sufficient information to develop a comprehensive predictive model. Details of all 13 feature descriptors are provided in Table 2. Here, the *iFeature* Python package^29^ was utilized to calculate all the 13 feature descriptors.

**Table 2:** Summary of 13 different sequence-based feature descriptors along with their corresponding description and dimension.

Order	Descriptors	Description	Dimension	References
1	AAC	Frequency of 20 amino acids	20	^46,47^
2	AAI	Different biochemical and biophysical properties extracted from the AAindex database	11	^46,48^
3	APAAC	Amphiphilic pseudo-amino acid composition	22	^49^
4	CTDC	Percentage of particular amino acid property groups	39	^46,50,51^
5	CTDD	Percentage of mutual conversion in amino acid properties	39	^46,50,51^
6	CTDT	Distribution of amino acid properties in sequences	195	^46,50,51^
7	DDE	Dipeptide deviation from expected mean	400	^52^
8	DPC	Frequency of 400 dipeptides	400	^47,53,54^
9	EAAC	Enhance amino acid composition	20	^52^
10	PAAC	Pseudo amino acid composition	21	^49^
11	PSSM_AAC	Traditional AAC from the primary sequence to the PSI-BLAST profile	20	^55^
12	PSSM_DP	Traditional PDC from the primary sequence to the PSI-BLAST profile	400	^55^
13	PSSM_COM	Position-specific scoring matrix composition	400	^55^

### Stacking ensemble learning framework of SCORPION

In this study, the stacked ensemble learning strategy was utilized to develop SCORPION for improving the prediction of PVPs. Unlike other ensemble learning strategies, this strategy enables an automatic integration of different ML classifiers in order to construct a single robust prediction model^23^. The stacked strategy has successfully achieve better performance as compared with its constituent baseline models^23,24,27,30,31^. The stacking strategy consists of two main steps, while the corresponding models at each step are referred to as baseline and meta models, respectively.

In the first step, the PVPs and non-PVPs in the training dataset were extracted by using 13 different feature encoding schemes from four different perspectives containing AAC, AAI, APAAC, CTDC, CTDD, CTDT, DDE, DPC, EAAC, PAAC, PSSM_AAC, PSSM_DP and PSSM_COM with corresponding dimensions of 20, 11, 22, 39, 39, 195, 400, 400, 20, 21, 20, 400 and 400, respectively^32–35^. Herein, we used the default *iFeature* parameter settings^29^ to generate APAAC and PAAC descriptors. Then, each feature descriptor was individually employ to train 10 different ML algorithms (KNN, RF, SVM, decision tree (DT), extremely randomized trees (ET), logistic regression (LR), multi-layer perceptron (MLP), naive Bayes (NB), partial least squares regression (PLS) and extreme gradient boosting (XGB)). To enhance the predictive performance, all ML classifiers were trained and optimized using the scikit-learn package in Python (version 0.22)^36^. Specifically, the optimal parameters of ET, LR, MLP, RF, SVM and XGB classifiers were carefully determined under the tenfold cross-validation procedure on the training dataset, where the search range is shown in Supplementary Table S1. In the case of the remaining ML classifiers, they were constructed by using their default parameters. Therefore, we obtained a total of 130 baseline models (10 MLs × 13 encodings).

In the second step, each baseline model provided us three types of features from three perspectives containing PF, class feature (CF) and the combination of PF and CF (PCF). The PF is based on the predicted probability scores to be PVPs which is in the range of 0–1. In case of the CF, the protein sequence *P* is labeled as 1 if its predicted probability scores is greater than 0.5, otherwise the protein sequence *P* is labeled as 0. As a result, the protein sequence *P* was represented to 130-D, 130-D and 260-D feature vectors for PF, CF and PCF, respectively. The PF, CF and PCF were considered as new feature vectors. RF algorithm was employed as the meta model (called mRF) to train the stacked model. As result, we obtained three different stacked models based on three new feature vectors (i.e. PF, CF and PCF). To improve the discriminative ability of the new feature vectors, we used a two-step feature selection strategy to optimize PF, CF and PCF feature vectors. At the first step, we used XGB classifier to rank the features in PF, CF and PCF. The XGB classifier is widely used in the feature importance analysis^23,37^. Using the XGB classifier, we constructed a ranking list of features with respect to their importance scores. Higher ranked features in this list are the most important features. At the second step, we constructed *n* different feature subsets containing the top ranked features ranging from top 5 to top 100 features with an interval of 5. Then, we inputted all feature subsets into mRF models and optimized the mRF models’ parameters using tenfold cross-validation scheme. The feature subset achieving the highest Matthews correlation coefficient (MCC) was considered as the optimal feature subset. The implementation of these classifiers in the two-step feature selection strategy is the same as used in our previous studies^18,31,38–41^

### Performance evaluation strategies

In order to examine the performance of our proposed predictor, we used five common statistical metrics including ACC, MCC, sensitivity (Sn) and specificity (Sp)^24,42^ as described follows:

1 $$\mathrm{ACC}=\frac{\mathrm{TP}+\mathrm{TN}}{\left(\mathrm{TP}+\mathrm{TN}+\mathrm{FP}+\mathrm{FN}\right)},$$

2 $$\mathrm{Sn}=\frac{\mathrm{TP}}{\left(\mathrm{TP}+\mathrm{FN}\right)},$$

3 $$\mathrm{Sp}=\frac{\mathrm{TN}}{\left(\mathrm{TN}+\mathrm{FP}\right)},$$

4 $$\mathrm{MCC}=\frac{\mathrm{TP}\times \mathrm{TN}-\mathrm{FP}\times \mathrm{FN}}{\sqrt{(\mathrm{TP}+\mathrm{FP})(\mathrm{TP}+\mathrm{FN})(\mathrm{TN}+\mathrm{FP})(\mathrm{TN}+\mathrm{FN})}},$$

where TP, TN, FP and FN represent the number of true positives, true negatives, false positive and false negatives, respectively. In addition, the area under the receiver operating characteristic (AUC) was employed as another statistical metric^39–41,43^.

## Results and discussion

### Performance evaluation between different classifiers and feature encodings

**Figure 2:**
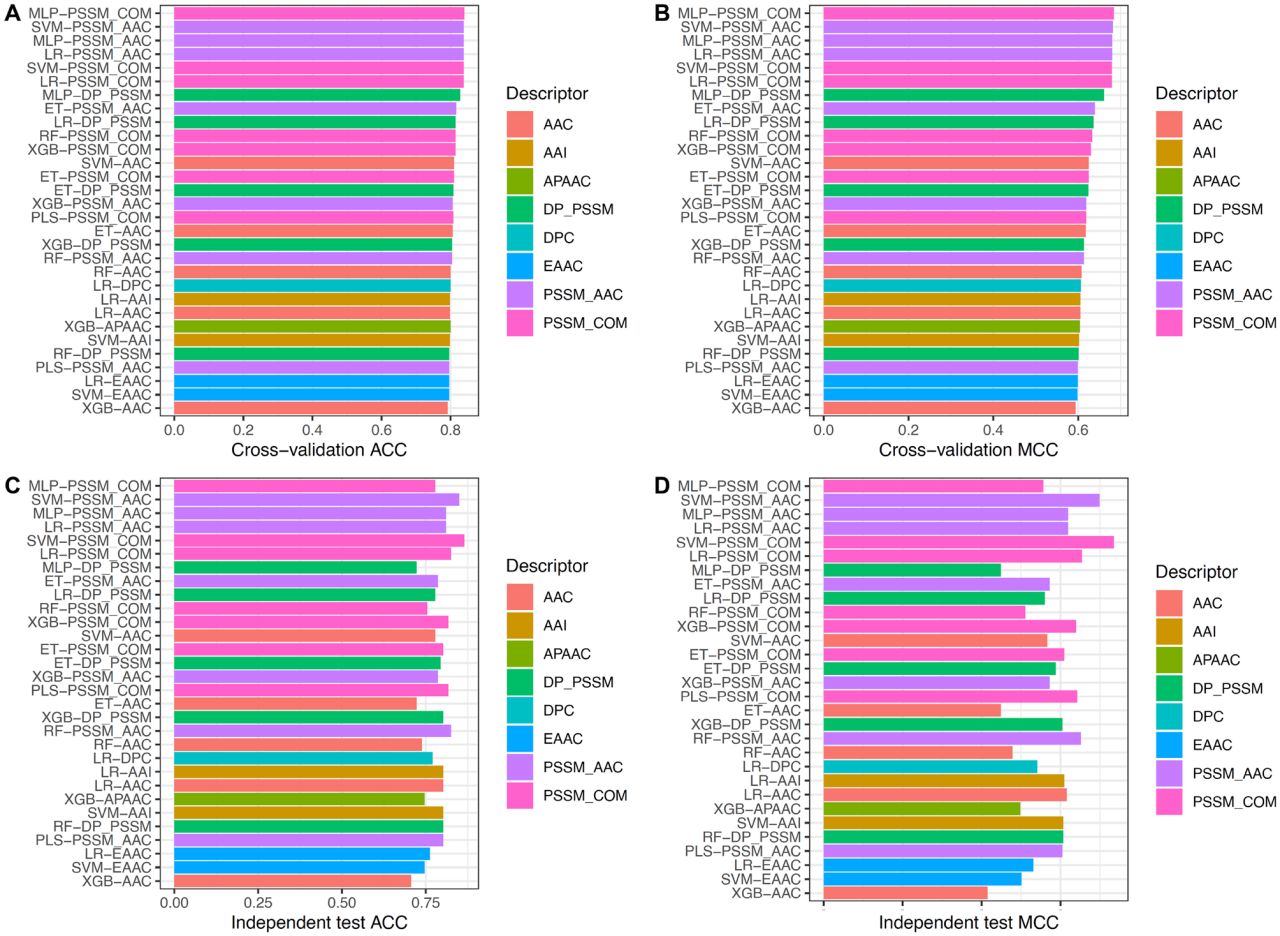
Performance evaluations of top 30 baseline models. (**A,B**) Cross-validation ACC and MCC of top 30 baseline models. (**C,D**) Independent test ACC and MCC of top 30 baseline models.

In this section, we investigated the effect of individual feature descriptor for PVP identification. Specifically, 13 different feature encoding from multiple perspectives (i.e. compositional information (AAC, APAAC, DDE, DPC, EAAC and PAAC), composition-transition-distribution information (CTDC, CTDD and CTDT), position-specific information (PSSM_AAC, PSSM_DP and PSSM_COM) and physicochemical properties (AAI)) were inputted to 10 different ML algorithms (DT, ET, KNN, LR, MLP, NB, PLS, RF, SVM and XGB) for developing 130 baseline models. We evaluated the predictive performance of the 130 baseline models with a default threshold of 0.5 by performing tenfold cross-validation and independent tests on the training and independent datasets, respectively. For convenience of discussion, Fig. 2 shows the performance of the 30 best-performing baseline models in the term of cross-validation MCC. In addition, the performance results for all the 130 baseline models are provided in Supplementary Tables S2 and S3.

From Fig. 2, Supplementary Tables S2 and S3, several observations can be made. First, PSSM_AAC descriptor was the most powerful one for PVP identification with average cross-validation AAC and MCC of 0.802 and 0.610, respectively. In the meanwhile, PSSM_COM and AAC descriptors performed well with the second and third highest average cross-validation MCC of 0.582 and 0.556, respectively. Second, SVM-based and LR-based classifiers outperformed other ML-based classifiers in terms of ACC (0.782–0.784), Sp (0.780–0.788), MCC (0.570–0.576) and AUC (0.849–0.850). Third, among all the 130 baseline models, the baseline model trained with MLP algorithm in conjunction with PSSM_COM descriptor (MLP-PSSM_COM) attained the best performance with cross-validation AAC and MCC of 0.840 and 0.684, while its ACC, MCC and AUC were 0.778, 0.556 and 0.859, respectively, as evaluated by the independent test. Taken together, the single feature-based models were not effective enough for PVP identification. On the other hand, the integration of variant ML classifier for constructing a single meta-predictor might improve the model’s performance.

### Performance evaluation of different stacked models

As mentioned in the “Materials and methods” section, we designed and developed three different stacked models based on three types of new feature representations consisting of PF (130D), CF (130D) and PCF (260D). Specifically, these three new feature representations were inputted to RF algorithm for developing three different mRF models. The performance comparison results amongst the three mRF models are provided in Tables 3 and 4. As can be seen, it is clear that PF and PCF feature vectors achieved better performance in terms of all performance metrics based on both tenfold cross-validation and independent tests. To further improve the discriminative ability of our new features, we utilized the two-step feature selection scheme to optimize PF, CF and PCF feature vectors. Herein, the feature selection scheme identified 50, 5 and 5 informative PFs, CFs and PCFs, respectively, for generating three optimal feature sets. Tables 3 and 4 shows that the three optimal feature sets attained a similar performance based on tenfold cross-validation test. In case of the independent test results, optimal PF feature vector outperformed other feature sets in terms of four out of five performance metrics (i.e. ACC, Sp, MCC and AUC). Particularly, ACC, Sp, MCC and AUC of optimal PF feature vector were 0.881, 0.952, 0.770 and 0.922, respectively (Table 4). The optimal PF feature vector consisted of the 50 informative features of PF. More details of the 50 informative features of PF were reported in Supplementary Table S4. Overall, we observed that the optimal PF feature vector was the most powerful feature for effectively capturing the key pattern of PVPs. For convenience of discussion, the mRF model trained with the optimal PF feature vector is referred herein as SCORPION.

**Table 3:** Cross-validation results for different feature representations using class and probabilistic information.

Features	Dimension	ACC	Sn	Sp	MCC	AUC
PF	130	0.858	0.840	0.876	0.722	0.914
CF	130	0.838	0.848	0.828	0.684	0.895
PCF	260	0.864	0.880	0.848	0.733	0.920
Optimal PF	50	0.868	0.884	0.852	0.743	0.920
Optimal CF	5	0.868	0.880	0.856	0.743	0.902
Optimal PCF	5	0.868	0.884	0.852	0.741	0.907

**Table 4:** Independent test results for different feature representations using class and probabilistic information.

Features	Dimension	ACC	Sn	Sp	MCC	AUC
PF	130	0.857	0.937	0.778	0.723	0.924
CF	130	0.817	0.746	0.889	0.642	0.892
PCF	260	0.857	0.778	0.937	0.723	0.925
Optimal PF	50	0.881	0.810	0.952	0.770	0.922
Optimal CF	5	0.802	0.794	0.810	0.603	0.859
Optimal PCF	5	0.873	0.841	0.905	0.748	0.891

### New feature representations improve the predictive performance

**Figure 3:**
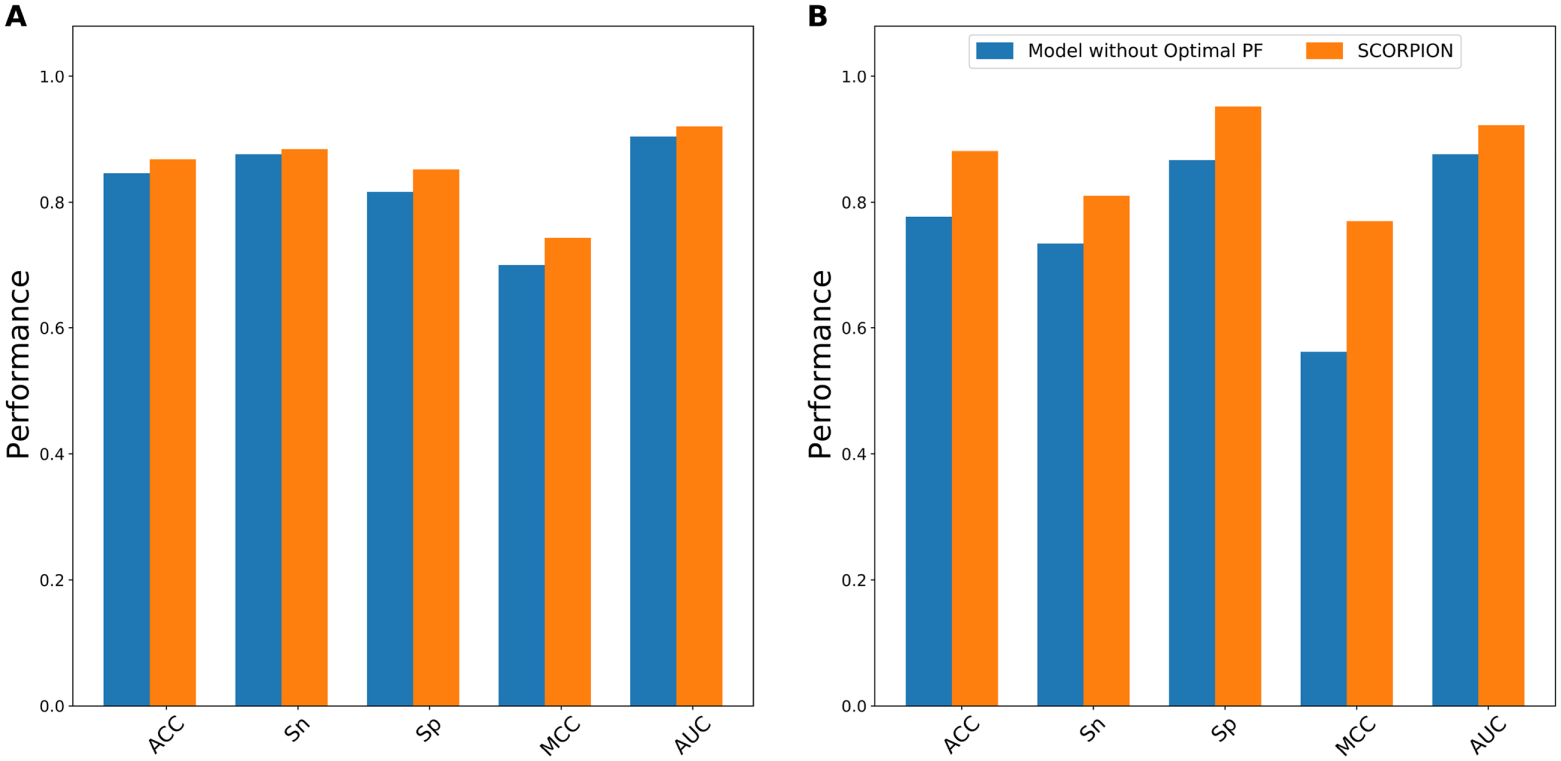
Performance comparison of SCORPION with the models without the optimal PF feature vector, as assessed by tenfold cross-validation (**A**) and independent test (**B**).

**Figure 4:**
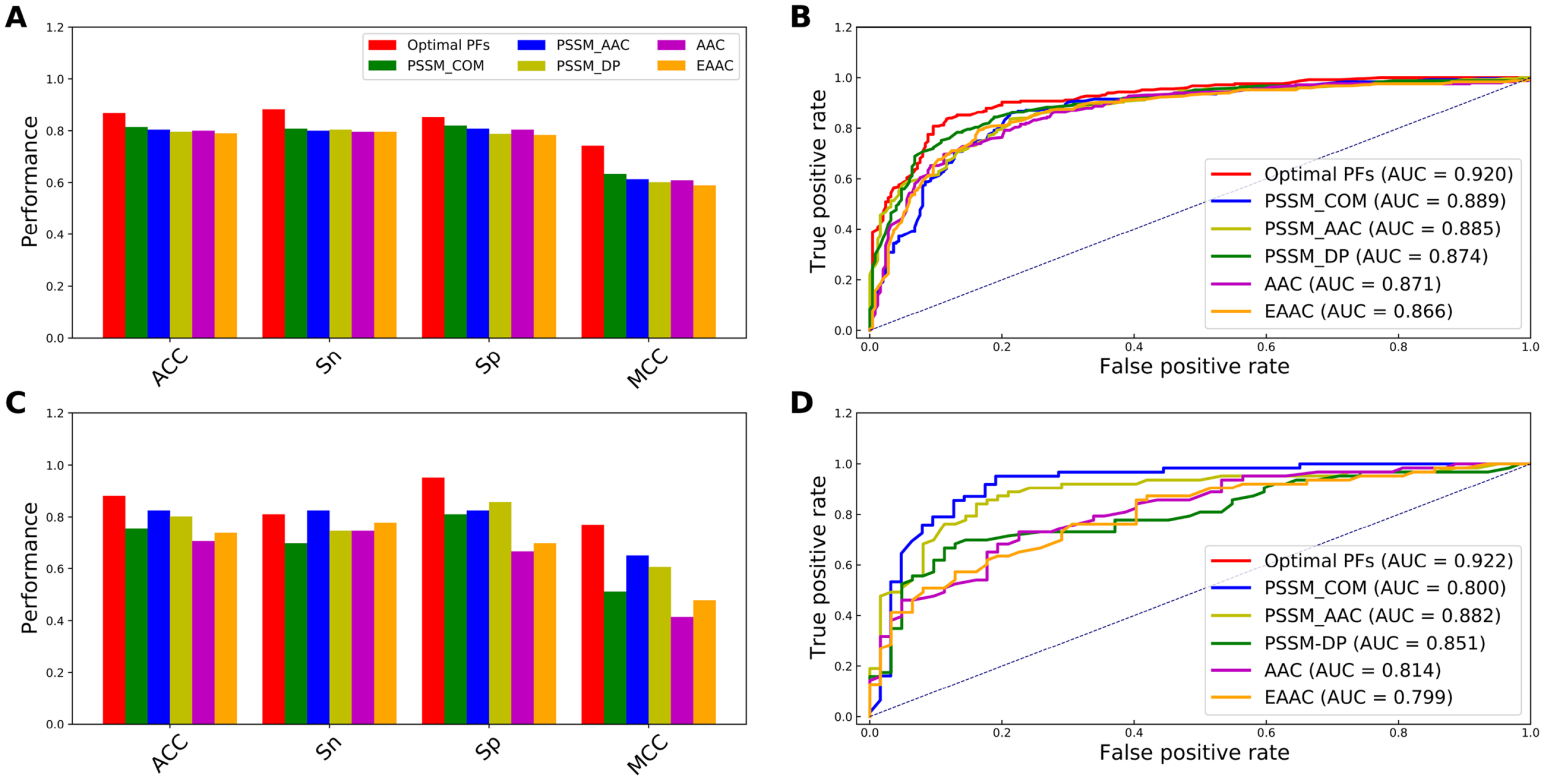
Performance comparison of the optimal PFs with the top five commonly used feature descriptors on the training (**A,B**) and independent tests (**C,D**). Prediction results of the optimal PFs with the top five commonly used feature descriptors in terms of ACC, Sn, Sp and MCC (**A,C**). ROC curves and AUC values of the optimal PFs with the top five commonly used feature descriptors (**B,D**).

To investigate whether the optimal PF feature vector is effective in improving the predictive performance, we investigate and performed three sets of comparative experiments as follows. First, we compared the performance of SCORPION (50D) with the model without the optimal PF feature vector (80D). Second, the performance of the optimal PF feature vector was compared with 13 different feature descriptors. Finally, we compared the performance of SCORPION with its constituent baseline models. The performance comparison results between SCORPION and other methods are provided in Figs. 3, 4, Supplementary Tables S5 and S6.

**Figure 5:**
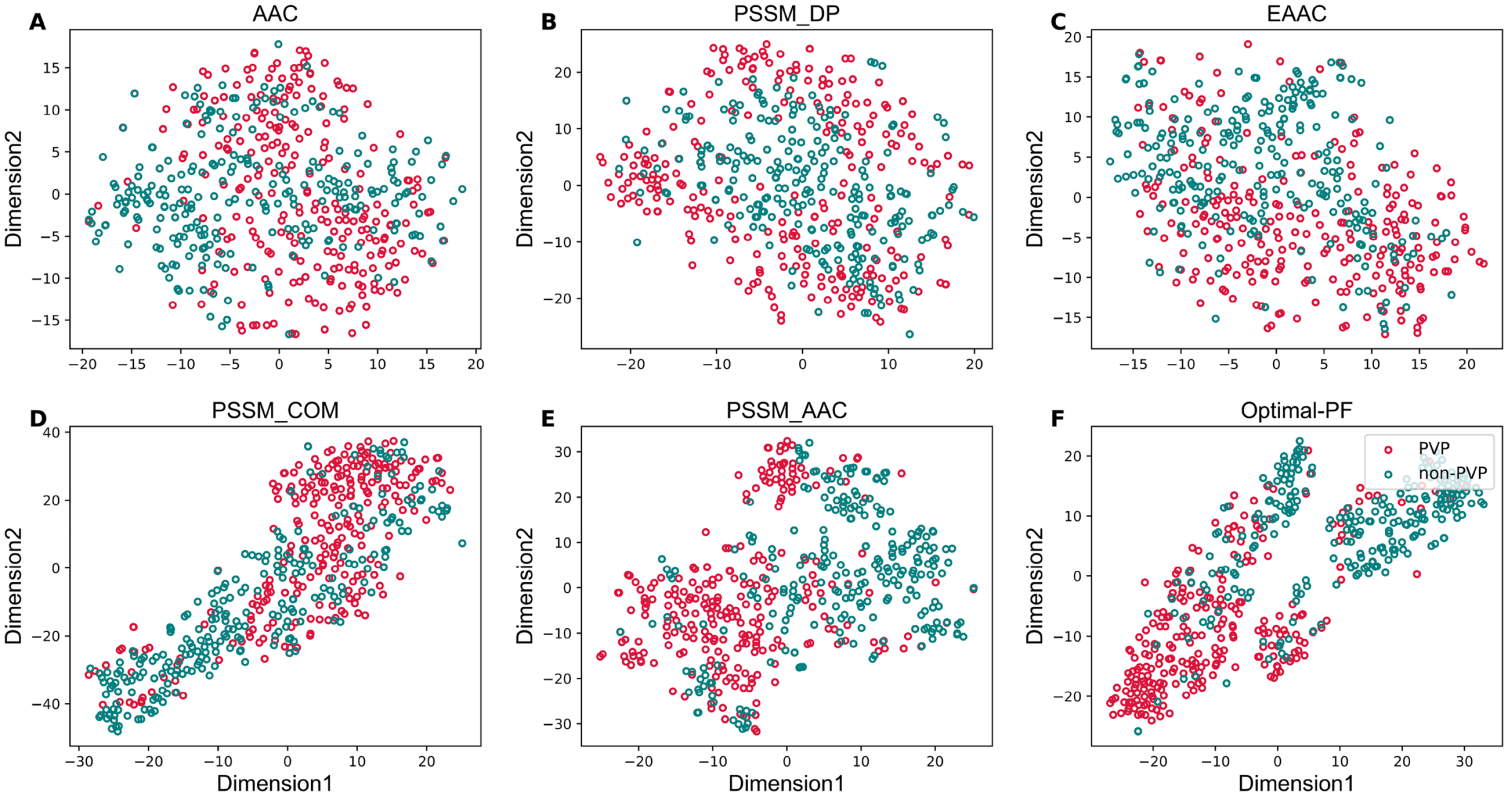
t-distributed stochastic neighbor embedding (t-SNE) distribution of positive and negative samples on the training dataset.

**Figure 6:**
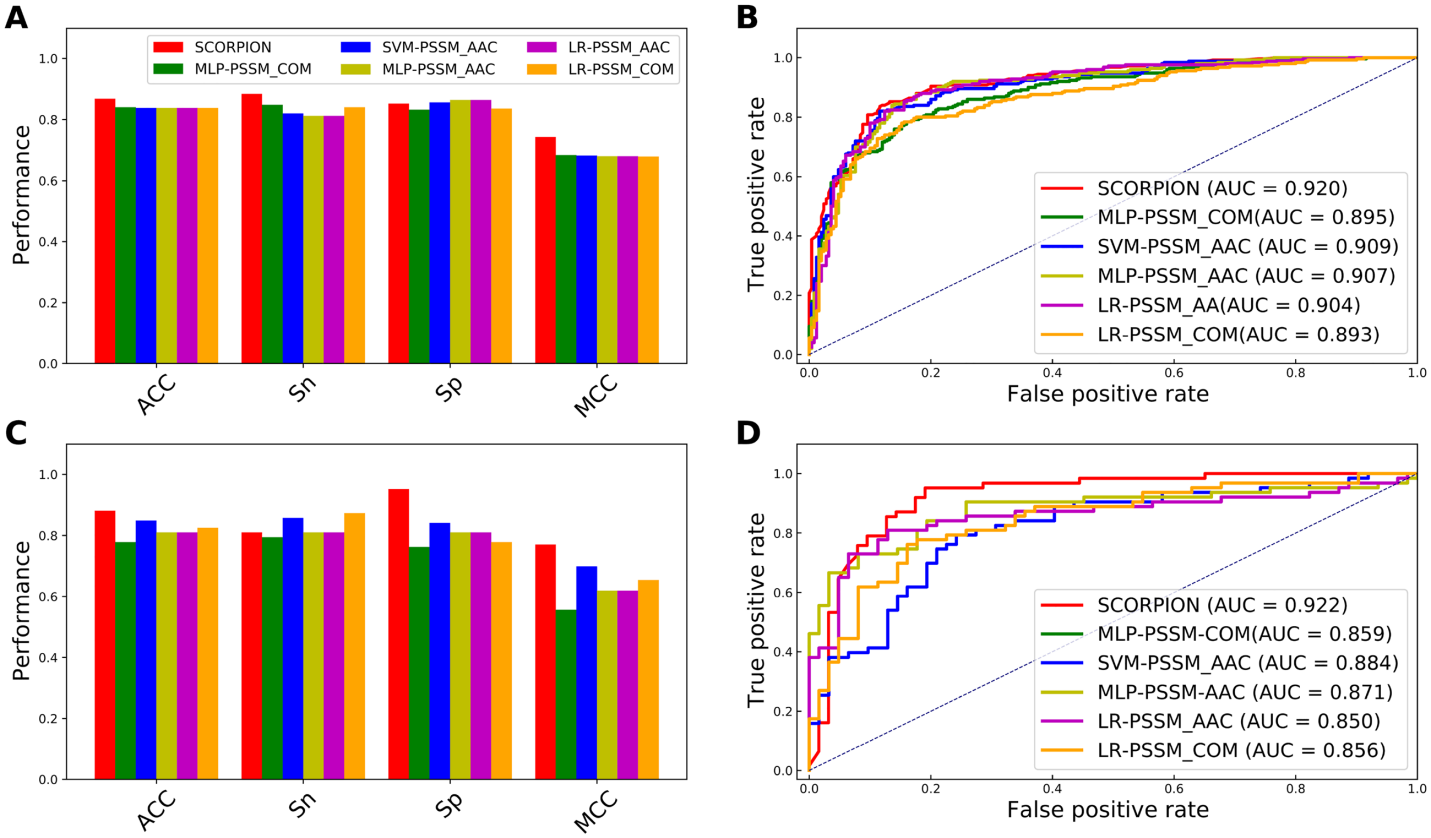
Performance comparison of SCORPION with the top five baseline models on the training (**A,B**) and independent tests (**C,D**). Prediction results of SCORPION and the top five baseline models in terms of ACC, Sn, Sp and MCC (**A,C**). ROC curves and AUC values of SCORPION with the top five baseline models (**B,D**).

From Fig. 3, we observe that SCORPION performed better than that of the model without the optimal PF feature vector in terms of all the five performance metrics on both the training and independent datasets. Impressively, ACC, Sn, Sp, MCC and AUC of SCORPION were 10.40%, 7.55%, 8.54%, 20.78% and 4.61%, respectively, higher than that of the model without the optimal PF feature vector on the independent dataset. After that, we compared the optimal PF feature vector with 13 different feature descriptors. As can be seen from Supplementary Tables S5 and S6, amongst 13 different feature descriptors, the five best-performing descriptors in terms of cross-validation MCC contained PSSM_COM, PSSM_AAC, AAC, PSSM_DP and EAAC. Here, we built RF classifiers with the five best-performing descriptors and evaluate the RF classifiers’ performance based on the tenfold cross-validation and independent tests. The performance comparison results between the optimal PF feature vector and these five best-performing descriptors are depicted in Fig. 4. In the meanwhile, Supplementary Table S5 shows that the highest cross-validation ACC and MCC of 0.868 and 0.743, respectively, were achieved by using the optimal PF feature vector, while PSSM_COM performed well with the second highest cross-validation ACC and MCC of 0.814 and 0.633, respectively. In case of the independent test results, the optimal PF feature vector significantly outperformed the second-best descriptor in terms of four out of five performance metrics (i.e. ACC, Sp, MCC and AUC). Specifically, the optimal PF feature vector’s ACC, Sp, MCC and AUC were 12.70%, 25.40%, 25.87% and 12.22%, respectively, higher than the second-best descriptor. In addition, we compared the distribution of the feature space of the optimal PF feature vector and the five best-performing descriptors on the training dataset by using the t-distributed stochastic neighbor embedding (t-SNE) based on the scikit-learn (version 0.22)^44,45^. Figure 5 shows six t-SNE plots representing their distributions between positive (red spots) and negative (green spots) samples in a 2D feature space. As can be seen, we notice that a clear separation between red and green spots was achieved in the feature space of the optimal PF feature vector. Finally, we compared the predictive performance of SCORPION against its constituent baseline models. Figure 2 shows that MLP-PSSM_COM performed well with the highest cross-validation ACC and MCC. As can be seen from Fig. 6, SCORPION attained the overall best performance as compared with MLP-PSSM_COM in terms of all performance metrics on both training and independent datasets. Remarkably, SCORPION’s ACC, Sp, MCC and AUC were 10.32%, 19.05%, 21.40% and 6.35%, respectively, higher than MLP-PSSM_COM. This confirmed that the optimal PF feature vector derived from the integration of variant ML classifier were beneficial for PVP identification and could improve the model’s predictive performance.

### Model interpretation

**Figure 7:**
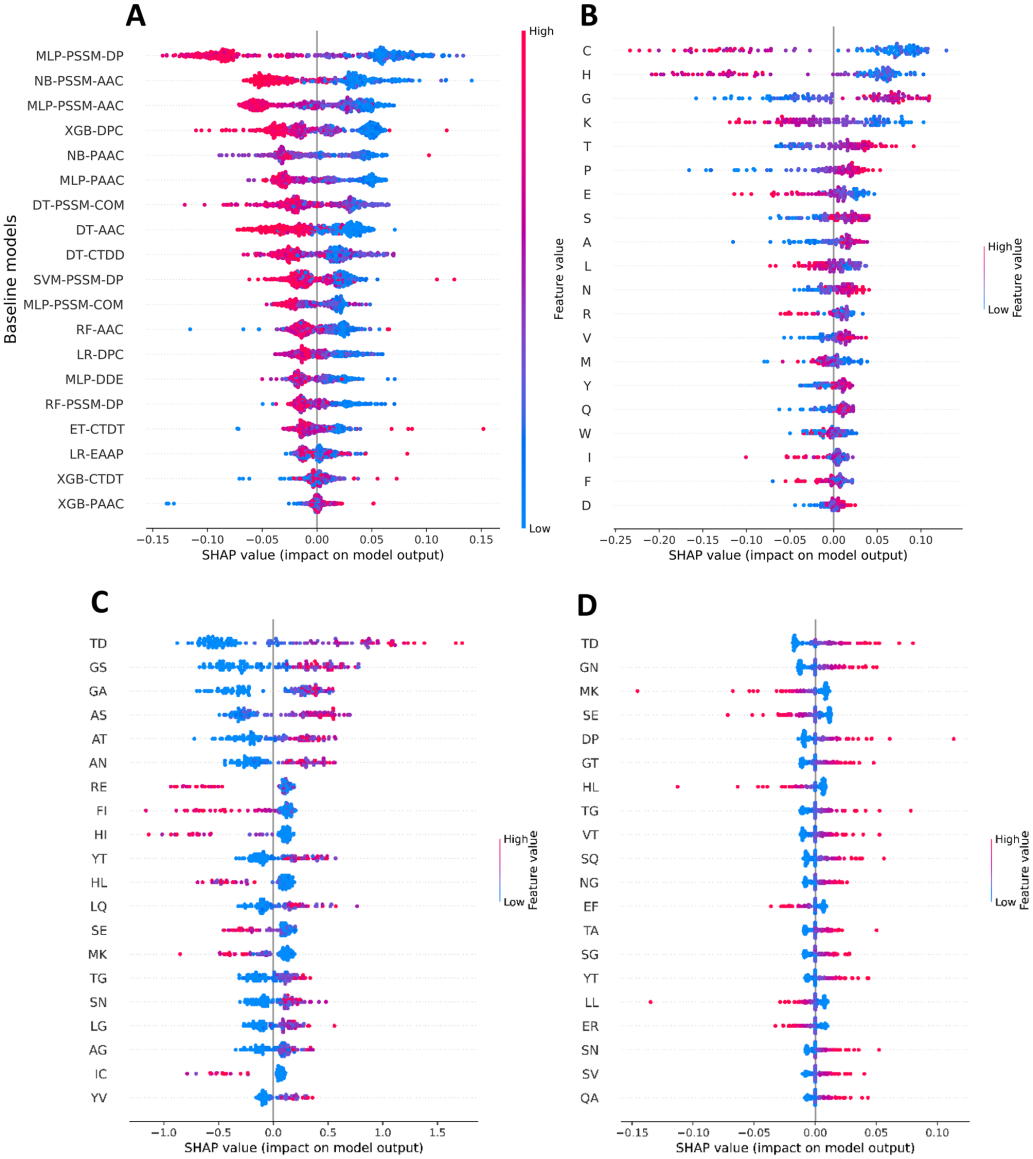
Feature importance from SCORPION (**A**) and selected three baseline models, where SHAP values represent the directionality of top features where negative and positive SHAP values influences the predictions toward PVPs and non-PVPs, respectively. SCORPION (**A**), RF-AAC (**B**), XGB-DPC (**C**) and LR-DPC (**D**).

In this section, we utilized the SHAP approach to analyze feature importance for SCORPION and three selected baseline models (i.e. RF-AAC, XGB-DPC and LR-XGB) for providing better understanding of these five models to generate their prediction outcomes. The impact of each feature on these three models’ prediction outcomes is illustrated in Fig. 7. To be specific, Fig. 7A–D show the top 20 PFs, top 20 amino acids and top 20 dipeptides respectively, based on SHAP values along with its directionality for each model, where the top 20 PFs were obtained from 20 top-ranked important features having the highest XGB classifier’s feature importance scores. Details of the top 20 PFs along with their feature importance scores are provided in Supplementary Table S7. It should be noted that negative and positive SHAP values drive the predictions as PVP and non-PVP classes, respectively, while the feature with the largest SHAP values is the most important. As seen in Fig. 7A, it is apparent that when the top five PFs of the five baseline models of MLP-PSSM_DP, NB-PSSM_AAC, MLP-PSSM_AAC, XGB-DPC and NB-PAAC had low SHAP values. Among these five baseline models, MLP-PSSM_AAC achieved the best performance in terms of cross-validation MCC (0.864). For a given unknown protein sequence *P*, it is predicted as PVP class if MLP-PSSM_AAC provides a low prediction probability, otherwise it is predicted as non-PVP class. From Fig. 7B, the five top-ranked informative amino acids based on SHAP values are Cys, His, Gly, Lys and Thr. Amongst these five top-ranked informative amino acids, Cys His and Lys exhibited low SHAP values, while Gly and Thr exhibited high SHAP values, suggesting that Cys His and Lys. From Fig. 7C,D, the seven top-ranked informative dipeptide based on SHAP values are TD, YT, HL, SE, MK, TG and SN.

### Comparison of SCORPION with conventional ML classifiers and existing methods

In this section, the same training and independent datasets established by Charoenkwan et al.^18^ were employed to assess and compare the predictive performance of SCORPION against and existing methods (i.e. PVPred, PVP-SVM, PVPred-SCM, Meta-iPVP and iPVP-MCV). The performance comparison results are shown in Tables 5 and 6. In case of the tenfold cross-validation results, SCORPION and iPVP-MCV achieved better performances than Meta-iPVP in terms of all performance metrics (Table 5). In addition, SCORPION secured the best predictive performance on the independent dataset, while iPVP-MCV attained the second-best performance value. Specifically, SCORPION significantly outperformed the compared existing method in terms of ACC, Sp and MCC, while iPVP-MCV achieved the best Sn (Table 6). In the meanwhile, SCORPION’s ACC, Sp and MCC were 4.80%, 17.44% and 9.88%, respectively, higher than iPVP-MCV. Altogether, our comparative results indicate that our predictor was able to attain the best predictive performance of PVP identification as compared to the existing methods.

**Table 5:** Cross-validation results of SCORPION and existing methods on the Charoenkwan’s dataset.

Methods^a^	ACC	Sn	Sp	MCC
Meta-iPVP	0.846	0.832	0.698	0.846
iPVP-MCV	0.864	0.876	0.728	0.864
SCORPION	0.868	0.852	0.743	0.868

^a^Performance of existing methods were obtained from the work iPVP-MCV^19^.

**Table 6:** Independent test results of SCORPION and existing methods on the Charoenkwan’s dataset.

Methods^a^	ACC	Sn	Sp	MCC
PVPred	0.730	0.892	0.663	0.505
PVP-SVM	0.746	0.816	0.701	0.505
PVPred-SCM	0.714	0.745	0.690	0.432
Meta-iPVP	0.817	0.889	0.746	0.642
iPVP-MCV	0.833	0.889	0.778	0.671
SCORPION	0.881	0.810	0.952	0.770

^a^Performance of existing methods were obtained from the work iPVP-MCV^19^.

The significant improvement of our predictor SCORPION can be characterized to three major reasons. First, our predictor was trained and optimized using an up-to-date dataset established by Charoenkwan et al.^18^ containing a larger number of PVPs and non-PVPs than other datasets. Second, our predictor combined variant sequence-based feature descriptors from different perspectives consisting of compositional information, composition-transition-distribution information, position-specific information and physicochemical properties. Third, the two-step feature selection scheme was utilized for identifying the most informative features that can help to precisely discriminate PVPs from non-PVPs.

## Conclusions

In this study, we introduced SCORPION, a novel, stacked, machine learning-based approach for accurate identification of PVPs. Specifically, SCORPION employed 13 different feature encoding schemes (categorized into four main groups) to encode PVPs and non-PVPs sequences and used 10 popular ML algorithms to build a pool of baseline models. These baseline models were then used to generate and construct the PF feature vector, which were considered as new feature representations. Finally, the optimal PF feature vector was optimized by using a two-step feature selection strategy and used this feature vector to develop the stacked model (SCORPION). Extensive benchmarking experiments show that SCORPION was effective and outperformed its constitute baseline models. In addition, when compared with five well-known existing methods (i.e. PVPred, PVP-SVM, PVPred-SCM, Meta-iPVP and iPVP-MCV) on the independent dataset, SCORPION achieved a superior predictive performance as compared the compared methods for PVP identification in terms of ACC (0.873), Sp (0.905), MCC (0.748) and AUC (0.891), thereby highlighting its effectiveness and generalizability. We anticipate that SCORPION will be a valuable tool for facilitating antibacterial drug discovery and development.

## Supplementary Information

Supplementary Tables.

## Data Availability

All the data used in this study are available at https://github.com/saeed344/SCORPION.
