# Supplementary material for: SCORPION is a stacking-based ensemble learning framework for accurate prediction of phage virion proteins
Source: Sci Rep. 2022 Mar 8;12:4106. doi: 10.1038/s41598-022-08173-5 (PMC8904530; doi:10.1038/s41598-022-08173-5)
Supplement: Supplementary file 1 — Supplementary Tables. [file 41598_2022_8173_MOESM1_ESM.docx]

**Table S1.** Hyperparameter search details for 10 different ML classifiers.

| **Method** | **Parameters** | **Range of parameters** |
| --- | --- | --- |
| DT | max_depth | Default |
| ET | n_estimators | [20, 50, 100, 200, 500] |
| KNN | number of neighbours | Default |
| LR | C | [0.001, 0.01, 0.1, 1, 10, 100] |
| MLP | hidden_layer_sizes | [50, 100, 300, 500] |
| NB | standard_deviation() | Default |
| PLS | #Components | Default |
| RF | n_estimators | [20, 50, 100, 200, 500] |
| SVM | Cost | [2^-4^–2^4^] in log_2_ steps |
| XGB | n_estimators | [20, 50, 100, 200, 500] |

Columns 2 and 3 represents the parameter name used in the Scikit-learn library and the range of parameter used to develop the model, respectively.

**Table S2** Cross-validation results of 130 baseline models developed using 10 different ML algorithms and 13 feature descriptors.

| **Descriptor** | **Method** | **ACC** | **Sn** | **Sp** | **MCC** | **AUC** |
| --- | --- | --- | --- | --- | --- | --- |
| AAC | DT | 0.684 | 0.704 | 0.664 | 0.375 | 0.689 |
|  | ET | 0.806 | 0.804 | 0.808 | 0.618 | 0.877 |
|  | KNN | 0.712 | 0.812 | 0.612 | 0.438 | 0.712 |
|  | LR | 0.798 | 0.820 | 0.776 | 0.605 | 0.869 |
|  | MLP | 0.788 | 0.784 | 0.792 | 0.580 | 0.846 |
|  | NB | 0.758 | 0.804 | 0.712 | 0.523 | 0.847 |
|  | PLS | 0.791 | 0.816 | 0.766 | 0.591 | 0.871 |
|  | RF | 0.800 | 0.796 | 0.804 | 0.608 | 0.871 |
|  | SVM | 0.810 | 0.796 | 0.824 | 0.625 | 0.870 |
|  | XGB | 0.792 | 0.812 | 0.772 | 0.594 | 0.852 |
| AAI | DT | 0.702 | 0.720 | 0.684 | 0.407 | 0.708 |
|  | ET | 0.758 | 0.768 | 0.748 | 0.522 | 0.831 |
|  | KNN | 0.698 | 0.764 | 0.632 | 0.400 | 0.698 |
|  | LR | 0.798 | 0.812 | 0.784 | 0.605 | 0.866 |
|  | MLP | 0.790 | 0.804 | 0.776 | 0.586 | 0.861 |
|  | NB | 0.752 | 0.756 | 0.748 | 0.510 | 0.794 |
|  | PLS | 0.748 | 0.712 | 0.784 | 0.503 | 0.820 |
|  | RF | 0.790 | 0.796 | 0.784 | 0.584 | 0.847 |
|  | SVM | 0.798 | 0.804 | 0.792 | 0.602 | 0.857 |
|  | XGB | 0.784 | 0.788 | 0.780 | 0.573 | 0.846 |
| APAAC | DT | 0.656 | 0.672 | 0.640 | 0.313 | 0.655 |
|  | ET | 0.726 | 0.720 | 0.732 | 0.456 | 0.783 |
|  | KNN | 0.606 | 0.832 | 0.380 | 0.240 | 0.606 |
|  | LR | 0.742 | 0.712 | 0.772 | 0.489 | 0.807 |
|  | MLP | 0.758 | 0.744 | 0.772 | 0.521 | 0.838 |
|  | NB | 0.674 | 0.556 | 0.792 | 0.363 | 0.748 |
|  | PLS | 0.687 | 0.695 | 0.680 | 0.378 | 0.715 |
|  | RF | 0.714 | 0.704 | 0.724 | 0.432 | 0.800 |
|  | SVM | 0.764 | 0.776 | 0.752 | 0.531 | 0.811 |
|  | XGB | 0.800 | 0.816 | 0.784 | 0.604 | 0.849 |
| CTDC | DT | 0.692 | 0.720 | 0.664 | 0.387 | 0.698 |
|  | ET | 0.766 | 0.780 | 0.752 | 0.539 | 0.826 |
|  | KNN | 0.690 | 0.764 | 0.616 | 0.388 | 0.690 |
|  | LR | 0.792 | 0.792 | 0.792 | 0.591 | 0.849 |
|  | MLP | 0.784 | 0.788 | 0.780 | 0.575 | 0.840 |
|  | NB | 0.754 | 0.752 | 0.756 | 0.515 | 0.805 |
|  | PLS | 0.760 | 0.756 | 0.764 | 0.525 | 0.820 |
|  | RF | 0.748 | 0.756 | 0.740 | 0.502 | 0.827 |
|  | SVM | 0.778 | 0.784 | 0.772 | 0.561 | 0.842 |
|  | XGB | 0.754 | 0.748 | 0.760 | 0.512 | 0.831 |
| CTDD | DT | 0.608 | 0.592 | 0.624 | 0.219 | 0.610 |
|  | ET | 0.690 | 0.716 | 0.664 | 0.383 | 0.747 |
|  | KNN | 0.608 | 0.680 | 0.536 | 0.220 | 0.608 |
|  | LR | 0.680 | 0.752 | 0.608 | 0.365 | 0.751 |
|  | MLP | 0.700 | 0.740 | 0.660 | 0.409 | 0.771 |
|  | NB | 0.674 | 0.800 | 0.548 | 0.363 | 0.734 |
|  | PLS | 0.675 | 0.747 | 0.604 | 0.357 | 0.734 |
|  | RF | 0.690 | 0.740 | 0.640 | 0.386 | 0.744 |
|  | SVM | 0.688 | 0.780 | 0.596 | 0.386 | 0.764 |
|  | XGB | 0.702 | 0.692 | 0.712 | 0.407 | 0.751 |
| CTDT | DT | 0.652 | 0.660 | 0.644 | 0.307 | 0.651 |
|  | ET | 0.758 | 0.760 | 0.756 | 0.524 | 0.801 |
|  | KNN | 0.670 | 0.748 | 0.592 | 0.346 | 0.670 |
|  | LR | 0.756 | 0.756 | 0.756 | 0.517 | 0.812 |
|  | MLP | 0.786 | 0.804 | 0.768 | 0.578 | 0.842 |
|  | NB | 0.726 | 0.788 | 0.664 | 0.460 | 0.796 |
|  | PLS | 0.756 | 0.760 | 0.752 | 0.517 | 0.808 |
|  | RF | 0.768 | 0.744 | 0.792 | 0.542 | 0.821 |
|  | SVM | 0.780 | 0.780 | 0.780 | 0.566 | 0.851 |
|  | XGB | 0.766 | 0.752 | 0.780 | 0.537 | 0.801 |
| DDE | DT | 0.632 | 0.648 | 0.616 | 0.268 | 0.632 |
|  | ET | 0.734 | 0.780 | 0.688 | 0.474 | 0.777 |
|  | KNN | 0.662 | 0.712 | 0.612 | 0.327 | 0.662 |
|  | LR | 0.770 | 0.740 | 0.800 | 0.543 | 0.858 |
|  | MLP | 0.728 | 0.716 | 0.740 | 0.461 | 0.800 |
|  | NB | 0.776 | 0.744 | 0.808 | 0.557 | 0.854 |
|  | PLS | 0.784 | 0.740 | 0.828 | 0.574 | 0.857 |
|  | RF | 0.712 | 0.776 | 0.648 | 0.432 | 0.813 |
|  | SVM | 0.780 | 0.772 | 0.788 | 0.565 | 0.847 |
|  | XGB | 0.752 | 0.760 | 0.744 | 0.510 | 0.825 |
| PSSM_DP | DT | 0.730 | 0.748 | 0.712 | 0.464 | 0.733 |
|  | ET | 0.808 | 0.816 | 0.800 | 0.624 | 0.882 |
|  | KNN | 0.712 | 0.764 | 0.660 | 0.431 | 0.712 |
|  | LR | 0.814 | 0.756 | 0.872 | 0.636 | 0.887 |
|  | MLP | 0.828 | 0.824 | 0.832 | 0.661 | 0.894 |
|  | NB | 0.626 | 0.348 | 0.904 | 0.305 | 0.640 |
|  | PLS | 0.759 | 0.587 | 0.931 | 0.553 | 0.853 |
|  | RF | 0.796 | 0.804 | 0.788 | 0.601 | 0.874 |
|  | SVM | 0.788 | 0.704 | 0.872 | 0.588 | 0.872 |
|  | XGB | 0.804 | 0.820 | 0.788 | 0.613 | 0.880 |
| DPC | DT | 0.604 | 0.648 | 0.560 | 0.210 | 0.608 |
|  | ET | 0.718 | 0.736 | 0.700 | 0.441 | 0.803 |
|  | KNN | 0.608 | 0.956 | 0.260 | 0.291 | 0.608 |
|  | LR | 0.800 | 0.844 | 0.756 | 0.606 | 0.858 |
|  | MLP | 0.734 | 0.800 | 0.668 | 0.476 | 0.786 |
|  | NB | 0.730 | 0.772 | 0.688 | 0.467 | 0.792 |
|  | PLS | 0.780 | 0.820 | 0.740 | 0.568 | 0.839 |
|  | RF | 0.714 | 0.688 | 0.740 | 0.435 | 0.792 |
|  | SVM | 0.788 | 0.764 | 0.812 | 0.584 | 0.849 |
|  | XGB | 0.730 | 0.740 | 0.720 | 0.463 | 0.803 |
| EAAC | DT | 0.700 | 0.696 | 0.704 | 0.402 | 0.703 |
|  | ET | 0.792 | 0.780 | 0.804 | 0.591 | 0.864 |
|  | KNN | 0.698 | 0.800 | 0.596 | 0.408 | 0.698 |
|  | LR | 0.796 | 0.820 | 0.772 | 0.599 | 0.866 |
|  | MLP | 0.786 | 0.808 | 0.764 | 0.576 | 0.836 |
|  | NB | 0.762 | 0.804 | 0.720 | 0.531 | 0.844 |
|  | PLS | 0.791 | 0.828 | 0.754 | 0.591 | 0.867 |
|  | RF | 0.790 | 0.796 | 0.784 | 0.589 | 0.866 |
|  | SVM | 0.796 | 0.784 | 0.808 | 0.598 | 0.860 |
|  | XGB | 0.762 | 0.784 | 0.740 | 0.530 | 0.846 |
| PAAC | DT | 0.694 | 0.680 | 0.708 | 0.391 | 0.699 |
|  | ET | 0.730 | 0.720 | 0.740 | 0.465 | 0.816 |
|  | KNN | 0.648 | 0.772 | 0.524 | 0.313 | 0.648 |
|  | LR | 0.744 | 0.732 | 0.756 | 0.493 | 0.820 |
|  | MLP | 0.786 | 0.784 | 0.788 | 0.575 | 0.849 |
|  | NB | 0.638 | 0.404 | 0.872 | 0.316 | 0.745 |
|  | PLS | 0.688 | 0.648 | 0.728 | 0.379 | 0.758 |
|  | RF | 0.742 | 0.716 | 0.768 | 0.489 | 0.819 |
|  | SVM | 0.756 | 0.744 | 0.768 | 0.515 | 0.821 |
|  | XGB | 0.770 | 0.744 | 0.796 | 0.546 | 0.840 |
| PSSM_AAC | DT | 0.730 | 0.780 | 0.680 | 0.465 | 0.731 |
|  | ET | 0.816 | 0.828 | 0.804 | 0.639 | 0.887 |
|  | KNN | 0.772 | 0.832 | 0.712 | 0.554 | 0.772 |
|  | LR | 0.838 | 0.812 | 0.864 | 0.680 | 0.904 |
|  | MLP | 0.838 | 0.812 | 0.864 | 0.680 | 0.907 |
|  | NB | 0.780 | 0.724 | 0.836 | 0.568 | 0.863 |
|  | PLS | 0.796 | 0.748 | 0.844 | 0.599 | 0.884 |
|  | RF | 0.804 | 0.800 | 0.808 | 0.613 | 0.885 |
|  | SVM | 0.838 | 0.820 | 0.856 | 0.682 | 0.909 |
|  | XGB | 0.806 | 0.800 | 0.812 | 0.619 | 0.885 |
| PSSM_COM | DT | 0.668 | 0.680 | 0.656 | 0.338 | 0.669 |
|  | ET | 0.810 | 0.792 | 0.828 | 0.625 | 0.883 |
|  | KNN | 0.738 | 0.864 | 0.612 | 0.494 | 0.738 |
|  | LR | 0.838 | 0.840 | 0.836 | 0.679 | 0.893 |
|  | MLP | 0.840 | 0.848 | 0.832 | 0.684 | 0.895 |
|  | NB | 0.714 | 0.804 | 0.624 | 0.440 | 0.805 |
|  | PLS | 0.808 | 0.816 | 0.800 | 0.619 | 0.870 |
|  | RF | 0.814 | 0.808 | 0.820 | 0.633 | 0.889 |
|  | SVM | 0.838 | 0.848 | 0.828 | 0.679 | 0.898 |
|  | XGB | 0.814 | 0.820 | 0.808 | 0.630 | 0.892 |

**Table S3** Independent test results of 130 baseline models developed using 10 different ML algorithms and 13 feature descriptors.

| **Descriptor** | **Method** | **ACC** | **Sn** | **Sp** | **MCC** | **AUC** |
| --- | --- | --- | --- | --- | --- | --- |
| AAC | DT | 0.643 | 0.667 | 0.619 | 0.286 | 0.653 |
|  | ET | 0.722 | 0.794 | 0.651 | 0.449 | 0.805 |
|  | KNN | 0.643 | 0.778 | 0.508 | 0.297 | 0.643 |
|  | LR | 0.802 | 0.905 | 0.698 | 0.616 | 0.888 |
|  | MLP | 0.730 | 0.778 | 0.683 | 0.462 | 0.787 |
|  | NB | 0.786 | 0.905 | 0.667 | 0.588 | 0.872 |
|  | PLS | 0.786 | 0.889 | 0.683 | 0.584 | 0.893 |
|  | RF | 0.738 | 0.778 | 0.698 | 0.478 | 0.814 |
|  | SVM | 0.778 | 0.873 | 0.683 | 0.566 | 0.865 |
|  | XGB | 0.706 | 0.762 | 0.651 | 0.415 | 0.796 |
| AAI | DT | 0.651 | 0.762 | 0.540 | 0.309 | 0.657 |
|  | ET | 0.802 | 0.810 | 0.794 | 0.603 | 0.816 |
|  | KNN | 0.659 | 0.746 | 0.571 | 0.322 | 0.659 |
|  | LR | 0.802 | 0.873 | 0.730 | 0.609 | 0.883 |
|  | MLP | 0.802 | 0.857 | 0.746 | 0.607 | 0.880 |
|  | NB | 0.794 | 0.841 | 0.746 | 0.590 | 0.843 |
|  | PLS | 0.746 | 0.778 | 0.714 | 0.493 | 0.854 |
|  | RF | 0.754 | 0.778 | 0.730 | 0.509 | 0.823 |
|  | SVM | 0.802 | 0.857 | 0.746 | 0.607 | 0.868 |
|  | XGB | 0.746 | 0.810 | 0.683 | 0.496 | 0.811 |
| APAAC | DT | 0.627 | 0.635 | 0.619 | 0.254 | 0.637 |
|  | ET | 0.738 | 0.762 | 0.714 | 0.477 | 0.761 |
|  | KNN | 0.516 | 0.762 | 0.270 | 0.036 | 0.516 |
|  | LR | 0.786 | 0.825 | 0.746 | 0.573 | 0.844 |
|  | MLP | 0.802 | 0.841 | 0.762 | 0.605 | 0.870 |
|  | NB | 0.706 | 0.635 | 0.778 | 0.417 | 0.774 |
|  | PLS | 0.690 | 0.730 | 0.651 | 0.382 | 0.806 |
|  | RF | 0.746 | 0.778 | 0.714 | 0.493 | 0.795 |
|  | SVM | 0.722 | 0.746 | 0.698 | 0.445 | 0.820 |
|  | XGB | 0.746 | 0.825 | 0.667 | 0.498 | 0.803 |
| CTDC | DT | 0.619 | 0.651 | 0.587 | 0.239 | 0.619 |
|  | ET | 0.762 | 0.810 | 0.714 | 0.526 | 0.790 |
|  | KNN | 0.619 | 0.762 | 0.476 | 0.248 | 0.619 |
|  | LR | 0.810 | 0.873 | 0.746 | 0.624 | 0.873 |
|  | MLP | 0.802 | 0.889 | 0.714 | 0.613 | 0.802 |
|  | NB | 0.746 | 0.762 | 0.730 | 0.492 | 0.859 |
|  | PLS | 0.778 | 0.810 | 0.746 | 0.557 | 0.862 |
|  | RF | 0.754 | 0.778 | 0.730 | 0.509 | 0.808 |
|  | SVM | 0.794 | 0.825 | 0.762 | 0.588 | 0.842 |
|  | XGB | 0.746 | 0.794 | 0.698 | 0.494 | 0.814 |
| CTDD | DT | 0.516 | 0.413 | 0.619 | 0.032 | 0.519 |
|  | ET | 0.683 | 0.667 | 0.698 | 0.365 | 0.729 |
|  | KNN | 0.484 | 0.524 | 0.444 | -0.032 | 0.484 |
|  | LR | 0.683 | 0.810 | 0.556 | 0.377 | 0.722 |
|  | MLP | 0.659 | 0.746 | 0.571 | 0.322 | 0.709 |
|  | NB | 0.675 | 0.825 | 0.524 | 0.366 | 0.739 |
|  | PLS | 0.653 | 0.778 | 0.525 | 0.313 | 0.705 |
|  | RF | 0.667 | 0.714 | 0.619 | 0.335 | 0.711 |
|  | SVM | 0.683 | 0.841 | 0.524 | 0.385 | 0.748 |
|  | XGB | 0.651 | 0.651 | 0.651 | 0.302 | 0.693 |
| CTDT | DT | 0.579 | 0.571 | 0.587 | 0.159 | 0.579 |
|  | ET | 0.714 | 0.762 | 0.667 | 0.431 | 0.770 |
|  | KNN | 0.595 | 0.730 | 0.460 | 0.198 | 0.595 |
|  | LR | 0.778 | 0.825 | 0.730 | 0.558 | 0.857 |
|  | MLP | 0.738 | 0.794 | 0.683 | 0.479 | 0.823 |
|  | NB | 0.754 | 0.825 | 0.683 | 0.513 | 0.839 |
|  | PLS | 0.738 | 0.778 | 0.698 | 0.478 | 0.841 |
|  | RF | 0.746 | 0.762 | 0.730 | 0.492 | 0.806 |
|  | SVM | 0.770 | 0.794 | 0.746 | 0.540 | 0.834 |
|  | XGB | 0.706 | 0.762 | 0.651 | 0.415 | 0.777 |
| DDE | DT | 0.603 | 0.571 | 0.635 | 0.207 | 0.603 |
|  | ET | 0.714 | 0.762 | 0.667 | 0.431 | 0.738 |
|  | KNN | 0.579 | 0.714 | 0.444 | 0.165 | 0.579 |
|  | LR | 0.778 | 0.762 | 0.794 | 0.556 | 0.879 |
|  | MLP | 0.770 | 0.762 | 0.778 | 0.540 | 0.853 |
|  | NB | 0.762 | 0.825 | 0.698 | 0.528 | 0.853 |
|  | PLS | 0.833 | 0.841 | 0.825 | 0.667 | 0.877 |
|  | RF | 0.754 | 0.825 | 0.683 | 0.513 | 0.800 |
|  | SVM | 0.817 | 0.810 | 0.825 | 0.635 | 0.849 |
|  | XGB | 0.722 | 0.762 | 0.683 | 0.446 | 0.803 |
| PSSM_DP | DT | 0.690 | 0.683 | 0.698 | 0.381 | 0.690 |
|  | ET | 0.794 | 0.825 | 0.762 | 0.588 | 0.828 |
|  | KNN | 0.738 | 0.810 | 0.667 | 0.481 | 0.738 |
|  | LR | 0.778 | 0.714 | 0.841 | 0.560 | 0.867 |
|  | MLP | 0.722 | 0.794 | 0.651 | 0.449 | 0.824 |
|  | NB | 0.611 | 0.365 | 0.857 | 0.255 | 0.622 |
|  | PLS | 0.762 | 0.635 | 0.889 | 0.542 | 0.838 |
|  | RF | 0.802 | 0.857 | 0.746 | 0.607 | 0.851 |
|  | SVM | 0.802 | 0.778 | 0.825 | 0.604 | 0.862 |
|  | XGB | 0.802 | 0.841 | 0.762 | 0.605 | 0.840 |
| DPC | DT | 0.571 | 0.540 | 0.603 | 0.143 | 0.576 |
|  | ET | 0.754 | 0.794 | 0.714 | 0.510 | 0.781 |
|  | KNN | 0.563 | 0.905 | 0.222 | 0.174 | 0.563 |
|  | LR | 0.770 | 0.810 | 0.730 | 0.541 | 0.867 |
|  | MLP | 0.706 | 0.730 | 0.683 | 0.413 | 0.808 |
|  | NB | 0.722 | 0.810 | 0.635 | 0.451 | 0.813 |
|  | PLS | 0.770 | 0.841 | 0.698 | 0.545 | 0.863 |
|  | RF | 0.730 | 0.667 | 0.794 | 0.464 | 0.773 |
|  | SVM | 0.770 | 0.730 | 0.810 | 0.541 | 0.824 |
|  | XGB | 0.738 | 0.778 | 0.698 | 0.478 | 0.782 |
| EAAC | DT | 0.603 | 0.619 | 0.587 | 0.206 | 0.608 |
|  | ET | 0.754 | 0.810 | 0.698 | 0.511 | 0.810 |
|  | KNN | 0.619 | 0.794 | 0.444 | 0.254 | 0.619 |
|  | LR | 0.762 | 0.841 | 0.683 | 0.531 | 0.886 |
|  | MLP | 0.762 | 0.873 | 0.651 | 0.537 | 0.802 |
|  | NB | 0.786 | 0.905 | 0.667 | 0.588 | 0.876 |
|  | PLS | 0.786 | 0.905 | 0.667 | 0.588 | 0.891 |
|  | RF | 0.706 | 0.746 | 0.667 | 0.414 | 0.799 |
|  | SVM | 0.746 | 0.841 | 0.651 | 0.501 | 0.845 |
|  | XGB | 0.698 | 0.762 | 0.635 | 0.400 | 0.778 |
| PAAC | DT | 0.627 | 0.651 | 0.603 | 0.254 | 0.632 |
|  | ET | 0.762 | 0.778 | 0.746 | 0.524 | 0.800 |
|  | KNN | 0.532 | 0.762 | 0.302 | 0.072 | 0.532 |
|  | LR | 0.786 | 0.810 | 0.762 | 0.572 | 0.864 |
|  | MLP | 0.794 | 0.810 | 0.778 | 0.588 | 0.866 |
|  | NB | 0.627 | 0.397 | 0.857 | 0.286 | 0.748 |
|  | PLS | 0.794 | 0.746 | 0.841 | 0.590 | 0.832 |
|  | RF | 0.786 | 0.794 | 0.778 | 0.572 | 0.822 |
|  | SVM | 0.794 | 0.794 | 0.794 | 0.587 | 0.845 |
|  | XGB | 0.746 | 0.778 | 0.714 | 0.493 | 0.825 |
| PSSM_AAC | DT | 0.722 | 0.730 | 0.714 | 0.445 | 0.722 |
|  | ET | 0.786 | 0.810 | 0.762 | 0.572 | 0.837 |
|  | KNN | 0.754 | 0.794 | 0.714 | 0.510 | 0.754 |
|  | LR | 0.810 | 0.810 | 0.810 | 0.619 | 0.850 |
|  | MLP | 0.810 | 0.810 | 0.810 | 0.619 | 0.871 |
|  | NB | 0.817 | 0.857 | 0.778 | 0.637 | 0.865 |
|  | PLS | 0.802 | 0.841 | 0.762 | 0.605 | 0.852 |
|  | RF | 0.825 | 0.825 | 0.825 | 0.651 | 0.882 |
|  | SVM | 0.849 | 0.857 | 0.841 | 0.699 | 0.884 |
|  | XGB | 0.786 | 0.810 | 0.762 | 0.572 | 0.857 |
| PSSM_COM | DT | 0.659 | 0.683 | 0.635 | 0.318 | 0.659 |
|  | ET | 0.802 | 0.873 | 0.730 | 0.609 | 0.835 |
|  | KNN | 0.706 | 0.889 | 0.524 | 0.443 | 0.706 |
|  | LR | 0.825 | 0.873 | 0.778 | 0.654 | 0.856 |
|  | MLP | 0.778 | 0.794 | 0.762 | 0.556 | 0.859 |
|  | NB | 0.659 | 0.730 | 0.587 | 0.321 | 0.739 |
|  | PLS | 0.817 | 0.889 | 0.746 | 0.642 | 0.860 |
|  | RF | 0.754 | 0.810 | 0.698 | 0.511 | 0.800 |
|  | SVM | 0.865 | 0.921 | 0.810 | 0.735 | 0.888 |
|  | XGB | 0.817 | 0.873 | 0.762 | 0.639 | 0.852 |

**Table S4**. List of 50 baseline models used for constructing our proposed stacked model (SCORPION).

| **Baseline models** |
| --- |
| MLP-PSSM_DP, DT-CTDD, NB-PAAC, RF-PSSM_DP, MLP-DDE, NB-PSSM_ACC, SVM-PSSM_DP, XGB-PAAC, ET-CTDT, XGB-DPC, DT-PSSM-Com, MLP-PSSM-Com, MLP-PAAC, XGB-CTDT, LR-EAAP, DT-AAC, MLP-PSSM_ACC, RF-AAC, LR-DPC, NB-CTDT, DT-PSSM_ACC, SVM-CTDC, SVM-EAAP, LR-PSSM-Com, SVM-AAC, SVM-APAAC, RF-EAAP, LR-DDE, LR-CTDC, LR-PSSM_ACC, NB-CTDD, NB-EAAP, XGB-PSSM-Com, RF-CTDD, PLS-PAAC, SVM-DPC, RF-CTDC, KNN-PSSM_ACC, DT-DDE, RF-DDE, PLS-CTDT, LR-CTDD, ET-DDE, NB-CTDC, DT-PSSM_DP, DT-CTDT, ET-PAAC, XGB-CTDC, PLS-APAAC, KNN-AAC, |

**Table S5**. Performances comparison between our new features and individual feature descriptors as evaluated by 10-fold cross-validation test

| **Feature** | **ACC** | **Sn** | **Sp** | **MCC** | **AUC** |
| --- | --- | --- | --- | --- | --- |
| Optimal PF | 0.868 | 0.884 | 0.852 | 0.743 | 0.920 |
| PSSM_COM | 0.814 | 0.808 | 0.820 | 0.633 | 0.889 |
| PSSM_AAC | 0.804 | 0.800 | 0.808 | 0.613 | 0.885 |
| AAC | 0.800 | 0.796 | 0.804 | 0.608 | 0.871 |
| PSSM_DP | 0.796 | 0.804 | 0.788 | 0.601 | 0.874 |
| EAAC | 0.790 | 0.796 | 0.784 | 0.589 | 0.866 |
| AAI | 0.790 | 0.796 | 0.784 | 0.584 | 0.847 |
| CTDT | 0.768 | 0.744 | 0.792 | 0.542 | 0.821 |
| CTDC | 0.748 | 0.756 | 0.740 | 0.502 | 0.827 |
| PAAC | 0.742 | 0.716 | 0.768 | 0.489 | 0.819 |
| DPC | 0.714 | 0.688 | 0.740 | 0.435 | 0.792 |
| APAAC | 0.714 | 0.704 | 0.724 | 0.432 | 0.800 |
| DDE | 0.712 | 0.776 | 0.648 | 0.432 | 0.813 |
| CTDD | 0.690 | 0.740 | 0.640 | 0.386 | 0.744 |

**Table S6**. Performances comparison between our new features and individual feature descriptors as evaluated by the independent test

| **Feature** | **ACC** | **Sn** | **Sp** | **MCC** | **AUC** |
| --- | --- | --- | --- | --- | --- |
| Optimal PF | 0.881 | 0.810 | 0.952 | 0.770 | 0.922 |
| PSSM_COM | 0.754 | 0.810 | 0.698 | 0.511 | 0.800 |
| PSSM_AAC | 0.825 | 0.825 | 0.825 | 0.651 | 0.882 |
| AAC | 0.738 | 0.778 | 0.698 | 0.478 | 0.814 |
| PSSM_DP | 0.802 | 0.857 | 0.746 | 0.607 | 0.851 |
| EAAC | 0.706 | 0.746 | 0.667 | 0.414 | 0.799 |
| AAI | 0.754 | 0.778 | 0.730 | 0.509 | 0.823 |
| CTDT | 0.746 | 0.762 | 0.730 | 0.492 | 0.806 |
| CTDC | 0.754 | 0.778 | 0.730 | 0.509 | 0.808 |
| PAAC | 0.786 | 0.794 | 0.778 | 0.572 | 0.822 |
| DPC | 0.730 | 0.667 | 0.794 | 0.464 | 0.773 |
| APAAC | 0.746 | 0.778 | 0.714 | 0.493 | 0.795 |
| DDE | 0.754 | 0.825 | 0.683 | 0.513 | 0.800 |
| CTDD | 0.667 | 0.714 | 0.619 | 0.335 | 0.711 |

**Table S7**. Top 20 informative PFs derived 20 baseline models along with their feature importance scores.

| Baseline models | Score |
| --- | --- |
| MLP-PSSM_DP | 0.2019 |
| DT-CTDD | 0.0538 |
| NB-PAAC | 0.0418 |
| RF-PSSM_DP | 0.0309 |
| MLP-DDE | 0.0286 |
| NB-PSSM_ACC | 0.0277 |
| SVM-PSSM_DP | 0.0215 |
| XGB-PAAC | 0.0214 |
| ET-CTDT | 0.0213 |
| XGB-DPC | 0.0206 |
| DT-PSSM-Com | 0.0204 |
| MLP-PSSM-Com | 0.0177 |
| MLP-PAAC | 0.0175 |
| XGB-CTDT | 0.0173 |
| LR-EAAP | 0.0132 |
| DT-AAC | 0.0123 |
| MLP-PSSM_ACC | 0.0118 |
| RF-AAC | 0.0114 |
| LR-DPC | 0.0108 |
| NB-CTDT | 0.0104 |
